# Pluripotent stem cells reveal the developmental biology of human megakaryocytes and provide a source of platelets for clinical application

**DOI:** 10.1007/s00018-012-0995-4

**Published:** 2012-04-24

**Authors:** Naoya Takayama, Koji Eto

**Affiliations:** Clinical Application Department, Center for iPS Cell Research and Application, Kyoto University, 53 Kawahara-cho, Shogoin, Sakyo-ku, Kyoto, 606-8507 Japan

**Keywords:** Human ESCs, Human iPSCs, Disease-specific iPSCs, ES/iPS-sac, MK, Platelet, Transfusion

## Abstract

Human pluripotent stem cells [PSCs; including human embryonic stem cells (ESCs) and induced pluripotent stem cells (iPSCs)] can infinitely proliferate in vitro and are easily accessible for gene manipulation. Megakaryocytes (MKs) and platelets can be created from human ESCs and iPSCs in vitro and represent a potential source of blood cells for transfusion and a promising tool for studying the human thrombopoiesis. Moreover, disease-specific iPSCs are a powerful tool for elucidating the pathogenesis of hematological diseases and for drug screening. In that context, we and other groups have developed in vitro MK and platelet differentiation systems from human pluripotent stem cells (PSCs). Combining this co-culture system with a drug-inducible gene expression system enabled us to clarify the novel role played by *c*-*MYC* during human thrombopoiesis. In the next decade, technical advances (e.g., high-throughput genomic sequencing) will likely enable the identification of numerous gene mutations associated with abnormal thrombopoiesis. Combined with such technology, an in vitro system for differentiating human PSCs into MKs and platelets could provide a novel platform for studying human gene function associated with thrombopoiesis.

## Introduction

Platelets are essential for hemostasis and thrombosis. They also play vital roles in wound repair, inflammation, neoangiogenesis, and tumor metastasis. In humans, approximately 1 × 10^11^ platelets are produced each day, presumably through the cytoplasmic fragmentation of megakaryocytes (MKs), their precursor cells, and production can be significantly increased on demand [1]. MK and platelet numbers are thought to be primarily controlled by thrombopoietin (TPO), whose serum levels may be regulated in part through a process entailing its binding to TPO/c-MPL (the thrombopoietin receptor) on platelets and MKs and its subsequent internalization and degradation (sponge theory) [2, 3].

Platelet production requires the commitment of hematopoietic stem cells (HSCs) to the MK lineage, followed by proliferation of the progenitors and terminal differentiation. During maturation, the MK precursor loses its capacity to divide but increases its ploidy through a process called endomitosis or endoreduplication, and also develops a unique membrane complex called the demarcation membrane system, as well as various types of granules, including lysosomes, dense granules, and α-granules. Fully mature MKs form an extensive internal demarcation membrane, which is continuous with the plasma membrane and serves primarily as a membrane reservoir for the formation of the precursors of cytoplasmic extensions called proplatelets [4, 5]. These sequential processes are controlled through the orchestrated activities of numerous signaling molecules, microRNAs, transcriptional factors, and their downstream target genes. Various gene-targeted mouse models have contributed to the characterization of the linkage between the machinery of MK maturation and platelet release, and the roles played by the transcriptional factors involved [6–11].

It has become evident, however, that some human diseases are not fully recapitulated in genetically engineered mouse models. In that regard, several groups, including ours, have developed in vitro MK and platelet differentiation systems from human pluripotent stem cells (PSCs) [12–17]. Human PSCs, which are easily accessible for controlled and specific genetic manipulation, are a promising tool for studying transcriptional regulation within the defined constraints of human MK development or cell signaling within platelets, which are anucleate and thus not amenable to direct gene manipulation. Moreover, patient-specific-induced pluripotent stem cells (iPSCs) enable one to investigate the pathogenesis of disease or test drugs using in vitro estimation assays based upon specific cell-traced differentiation, without the need for human subjects. This review focuses on the study of human thrombopoiesis using in vitro differentiation systems for human PSCs.

## The study of human thrombopoiesis in vitro

### Sources of MKs and platelets

The rarity of MKs within bone marrow limits the use of bone marrow specimens for studies of megakaryopoiesis and platelet generation. For this reason, human thrombopoiesis has largely been studied using in vitro differentiation systems developed from human primary hematopoietic stem/progenitor cells derived from cord blood, bone marrow, and mobilized peripheral blood-derived CD34+ cells [18–21]. These systems have enabled each developmental step in the process of megakaryopoiesis to be resolved and examined, and have also contributed to our understanding of human platelet biogenesis. However, primary human hematopoietic cells are heterogeneous, reflecting the differences in their genetic backgrounds, which can lead to a lack of consistency in the results. Moreover, while platelets are not amenable to genetic manipulation, it is possible to manipulate MKs to investigate the molecules expressed in platelets, but the efficiency of genetic transduction in MKs and their progenitors is not high.

On the other hand, we previously showed the utility of an in vitro MK differentiation system derived from genetically manipulated mouse ESCs [22], which can be easily expanded and provides relatively homogeneous cells. This strategy also seemed applicable to the study of human thrombopoiesis using human PSCs, prompting us to establish an in vitro differentiation system with human PSCs that enables genetic manipulation for investigating human thrombopoiesis.

### In vitro generation of MKs and platelets from human PSCs

The culture methods needed to maintain the pluripotency of mouse ESCs are now well established and were improved by the finding that administration of two inhibitor (2i) reagents (MAP kinase inhibitor and GSK3β inhibitor) stabilizes the ground state/naive state [23]. In addition, differentiation protocols are being developed for application to a variety of cells and tissues, and several groups have reported producing platelets and/or MKs in vitro [22, 24, 25]. The first report of MK generation from human ESCs was presented by Gaur et al. [26]. They differentiated human ESCs on OP9 cells, a bone marrow stromal cell line derived from mice lacking macrophage colony stimulating factor [27]. OP9 cells are useful feeder cells and support differentiation of both mouse and human ESCs into mature hematopoietic cells in vitro. Gaur’s group observed that after 15–17 days of co-culture in the presence of TPO, ESC-derived MKs began to express specific surface antigens, including two lineage markers, cluster of differentiation (CD) 41a (CD41a; integrin αIIb) and CD42b (GPIbα), but no platelets were detected in this system [26]. We also failed to generate large numbers of platelet-like particles from human ESCs by this basic protocol.

By testing various combinations of feeder cells, cytokines, and culture periods, we were able to develop a novel in vitro differentiation system in which platelet-generating MKs were generated from human ESCs [12]. When we co-cultured human ESCs with 10T1/2 cells, a highly stable fetal mesenchymal cell line derived from a C3H mouse strain [28], in the presence of vascular endothelial growth factor (VEGF), unique sac-like structures derived from the ESCs (ES-sacs) appeared on day 14 of culture.

**Fig. 1:**
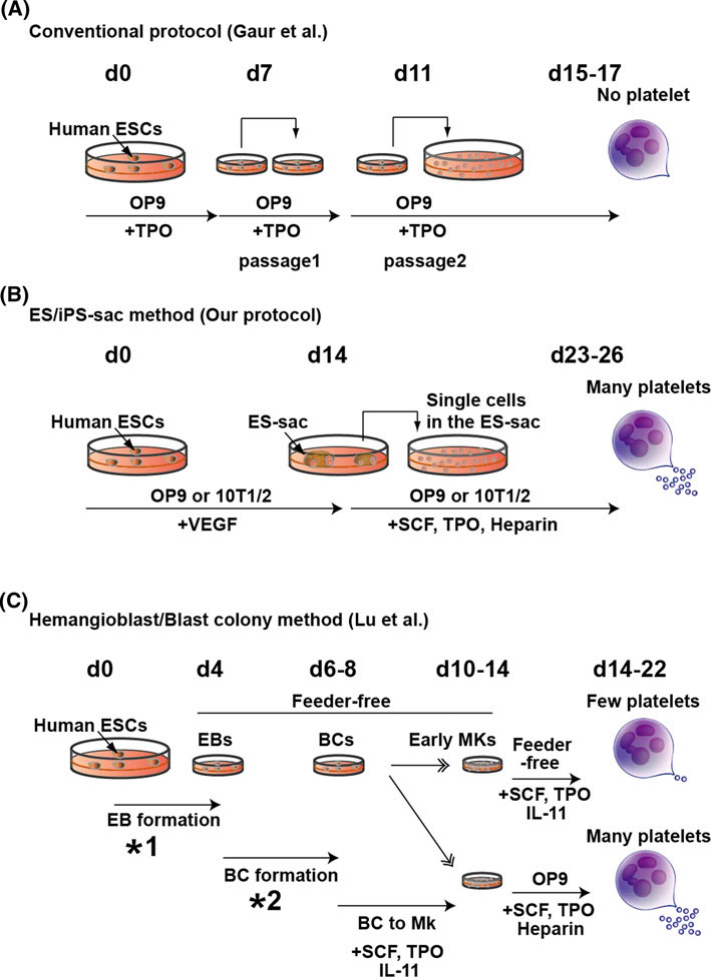
Schematic diagrams of culture systems generating megakaryocyte and platelet from human pluripotent stem cells. **a** Conventional method (multi-round replating); hESCs are differentiated on OP9 stromal cells. On days 7 and 11, single cells derived from differentiated human ESC colonies are transferred onto fresh OP9 cells and further cultivated up to 17 days. In this culture system, platelets were hardly produced as reported. **b** ES/iPS-sac method; hESCs are co-cultured with 10T1/2 or OP9 stromal cells. On day 14 of culture, ESC/iPSC-derived sacs (ES/iPS-sac) are collected by using cell scraper and filtered with cell strainer to concentrate hematopoietic progenitors. The hematopoietic progenitors are then transferred onto fresh 10T1/2 or OP9 stromal cells and cultivated up to day 26. Megakaryocytes can be matured for producing platelets efficiently in this protocol. **c** Hemangioblast/Blast colony (BC) method; hESCs are transferred to ultra-low attached culture dishes in serum-free condition with cytokine combination (*1) for ~4 days leading to embryoid body (EB) formation. Single cells derived from EBs are again transferred to ultra-low attached culture dishes in serum-free condition with cytokine combination (*2) and 1% methylcellulose for 2–4 days towards promotion of blast colonies (BCs). BCs are differentiated to MK lineage cells in the presence of SCF, TPO, IL-11 for 4–6 days. These steps are all feeder-free conditions. Platelet-generation stage requires OP9 stromal cells as evidenced by efficient yield of platelets but not without OP9 stromal cells. (*1) BMP4, VEGF, SCF, TPO, and FLT3L, (*2) BMP4, VEGF, SCF, TPO, EPO, FLT3L, G-CSF, GM-CSF, and IL-6

While it is well known that conventional ESC differentiation protocol with feeder cells by Gaur et al. [26] require the reseeding procedures both on day 7 and 11 (Fig. 1a), there was no necessity of reseeding procedure for up to 14 days on the appearance of ES-sac structure in our protocol (Fig. 1b).

These ES-sacs, particularly their external layer, expressed several endothelial markers, including CD31, CD34, and VEGF-receptor 2. ES-sac also contained CD34+/CD43+/CD45+ hematopoietic progenitors inside that exhibited hematopoietic colony-forming potential in semisolid culture and differentiated into a variety of mature cells, i.e., not only MKs capable of releasing platelets, but also erythrocytes, lymphocytes, macrophages, and granulocytes, under conditions towards specific lineage commitment.

On the other hand, hESC-derived colonies that did not form ES-sacs failed to differentiate into hematopoietic cells, indicating that the ES-sac concentrated multipotent hematopoietic progenitors inside.

Early hematopoietic cells reportedly originate from hemangioblasts [29], which are a common precursor of both vascular endothelial cells and hematopoietic cells. ES-sacs may mimic the early hematopoietic organ, within which hematopoiesis and vasculogenesis occur simultaneously, and may also contain hematopoietic niche circumstances for hematopoietic stem cells (HSCs) and/or hematopoietic progenitor cells (HPCs).

Co-culture for an additional 7–11 days in the presence of 100 ng/ml TPO, 50 ng/ml stem cell factor (SCF) and 25 U/ml heparin promotes differentiation of the hematopoietic progenitors within the ES-sacs into MKs [12, 13]. Of the cells, 50–60 % consistently express CD41a, CD42a, and CD42b, which are essential and specific cell-surface molecules expressed on MKs and platelets [30, 31]. During MK maturation, a broad range of internal membrane systems, granules, and organelles are assembled [32–34], and the mature MKs contain an extensive internal demarcation membrane that serves as a membrane reservoir for later formation of proplatelets. In addition, an open canalicular system (OCS) for granule release is formed prior to the initiation of proplatelet assembly, so that when platelets are stimulated, the granules are released via the OCS [35]. We found a large number of proplatelet-forming MKs from day 22 to day 26 of culture, suggesting a successful development of demarcation membrane system (DMS) for platelet release and yield.

We also confirmed that various types of human iPSCs can be differentiated into mature MKs that release platelets in vitro [13], and we are presently confident that the ES/iPS-sac method is a consistent and stable system for creating hematopoietic cells in vitro.

To establish xeno-free platelet generation system, Lu et al. have recently developed a unique feeder- and animal serum-free culture system in which ‘hemangioblast’ can be efficiently induced into hematopoietic progenitors [36], which in turn create erythrocytes [37], MKs, and platelets [15]. On the other hand, in such a system, adequate platelet production (yield) still must require known feeder cells OP9 mouse stromal cells, indicating that platelet release should be dependent upon feeder cells per se and/or critical factor(s) from feeder cells (Fig. 1c).

### Development and maturation of MKs derived from human PSCs

The use of human HSCs has enabled the process of MK development to be characterized in detail [33]. The most primitive MK progenitors are the highly proliferative potential-colony-forming unit-MKs (HPP-CFU-MKs) and the burst-forming unit-MKs (BFU-MKs). BFU-MKs are capable of producing a more differentiated MK progenitor, the colony-forming unit-MK (CFU-MK), which can then differentiate into mature MKs that increase in size and ploidy through endomitosis. The maturation of MKs from HSCs is associated with changes in the expression of CD antigens, whereas MK progenitors (HPP-CFU-MK, BFU-MK, and CFU-MK) and immature MKs express CD34 [38], more mature MKs are negative for CD34 expression. The most widely used markers for examining MK differentiation are CD41a/CD61 and CD42 (GPIb/IX/V complex; the vWF receptor). CFU-MKs are the first MK lineage to have been identified based on a distinct surface phenotype that expresses CD41a/CD61 antigen. CD42 is expressed slightly later than CD41a/CD61 [38]; however, the expression of both correlate with MK maturity. Thus, during megakaryopoiesis from human HSCs, the CD34+CD41a+CD42+ and CD34−CD41a+CD42+ phenotypes represent more mature MKs, while CD34+CD41a+CD42− cells are intermediate, not fully mature, MKs.

To clarify the relationship between the development of MKs derived from human PSCs and the changes in CD antigens, we isolated and divided hematopoietic progenitors from ES-sacs into CD34+/CD41a−, CD34+/CD41a+, CD34–/CD41a+, and CD34−/CD41a− subpopulations, and then allowed them to differentiate into MKs on 10T1/2 feeder cells or used RT-PCR to assess their gene expression. Most CD34+/CD41a+ and CD34−/CD41a+ cells should be differentiated into CD41a+/CD42b (GPIbα)+ mature MKs, indicating both populations were committed to MK lineage.

On the other hand, 40 % of hematopoietic cells derived from the CD34+/CD41a− population were MKs. This population generated multi-lineage myeloid colonies, such as erythrocyte, MKs, macrophage, or granulocytes, in colony-forming assay, indicating that the CD34+/CD41a− population contains the potential for differentiation into both megakaryocytic and other lineages. Using RT-PCR, we confirmed that CD34+/CD41a− progenitors can develop into CD41a+ populations. In addition, the CD34+/CD41a+ cells, but not CD34+/CD41a− cells, also expressed GATA-1, FOG-1, Fli-1, and NF-E2, which are required for megakaryopoiesis [39]. These findings are consistent with the developmental behavior observed during megakaryopoiesis from HSCs within bone marrow or cord blood [40, 41].

### Characterization of human ESC/iPSC-derived platelets in vitro

When we examined the surface markers of human ESC/iPSC-derived platelet-like particles using flow cytometry with the same forward- and side-scatter gates suitable for fresh adult human platelets, we found that the culture supernatants contained CD41a+ particles, which also expressed other functional receptors normally found on platelets, including CD42a (GPIX), CD42b (GPIbα), and GPVI. About half of the CD41a+ platelets generated in vitro lost CD42b (GPIbα) due to cleavage of its extracellular N-terminal region by a disintegrin and metalloproteinase (ADAM) 17 (also referred to as tumor necrosis factor α converting enzyme, TACE) [42], though GPIbα could be restored by addition of a metalloproteinase inhibitor to the culture [13].

Electron microscopic examination of the cytosolic structures of human ESC/iPSC-derived platelets revealed their morphology to be similar to that of human peripheral blood-derived platelets, with normal microtubules, granules, and OCS. These platelets also displayed integrin αIIbβ3 (CD41a/CD61 complex) activation, which is essential for platelet aggregation, and they spread in response to the physiological agonists ADP or thrombin. Human ESC/iPSC-derived platelets thus appear structurally and functionally intact in vitro, but the most important function of platelets in vivo is hemostasis and thrombus formation under flow conditions. Earlier in vivo imaging was capable of detecting only mass platelet aggregation [43]. However, to visualize the behavior of individual platelets upon initiation of adhesion to an injured vessel wall and the subsequent steps in thrombus formation under flow conditions within vessels, we recently developed a novel in vivo imaging system that employs high-spatiotemporal resolution confocal laser microscopy [44, 45]. With our system, intravenous administration of hematoporphyrin followed by laser exposure induces rapid formation of a microthrombus. In these experiments, iPSC-platelets stained with tetramethylrhodamine ethyl ester, a membrane-permeant, cationic, red-orange fluorescent dye that is readily sequestered by active mitochondria, were transfused into a NOG (NOD/Shi-scid, IL-2Rγnull) mouse with thrombocytopenia, where red staining then indicated living iPSC-platelets. Following laser-induced vessel injury, we clearly observed that iPSC-platelets initially adhered to the injured vessel wall, coordinating with host platelets, and that this process ultimately led to thrombus formation and vessel occlusion, which suggests the iPSC-derived platelets are functionally intact in vivo [13]. Similarly, using a conventional imaging system that enabled them to detect mass aggregations, Lu et al. [15] observed that human ESC-derived platelets contributed to thrombus formation in living mice. Collectively then, these findings indicate that platelets generated from human PSCs in vitro share many, if not all, of the characteristics of human peripheral blood-derived platelets. Using human iPSC specimens, we also confirmed that human iPSCs created from dermal fibroblasts through artificial reprogramming could be differentiated in vitro into functional platelets that could serve to supply HLA-identical platelets, or perhaps the use of disease-specific iPSCs could provide new information able to serve as a platform for the study of abnormal thrombopoiesis.

### In vitro analysis of the genes for platelet biogenesis using PSCs with gene manipulation

Megakaryopoiesis is a complex process governed by the orchestrated activities of numerous molecular mediators, especially transcriptional factors, signaling molecules, microRNAs, and the products of their various target genes. Several gene-targeted mouse models have provided important information about thrombopoiesis, but the generation of gene-targeted mice is both time-consuming and expensive; moreover, it is sometimes embryonically lethal before the onset of hematopoiesis, which makes it difficult to analyze the MK lineage. In that context, several groups, including ours, previously showed the advantages of in vitro differentiation of MKs from mouse ESCs over gene-targeted mouse models for analysis of functional molecules in MKs and platelets [22, 46–48].

The Wiskott-Aldrich syndrome protein (WASp) homolog Wave (WASp family Verprolin-homologous protein) functions downstream of Rac, a member of the Rho family of small GTPases, and plays a pivotal role in lamellipodia formation in cells. Wave2-null mice die by embryonic day 11.5 or 12.5 due to a defect in vascular development. We therefore generated Wave2−/− mouse ESCs from which we then derived MKs using our in vitro differentiation culture system. The Wave2−/− MKs generated in this way showed severely impaired terminal differentiation, platelet production, and agonist-induced peripheral lamellipodia formation on fibrinogen. We also confirmed that using small interfering RNA (siRNA) to knock down Abl-interactor (Abi)1, which forms a complex with Wave2, produces a phenotype that is striking similar to Wave2−/− MKs; that is, both their maturation and integrin αIIbβ3-mediated spreading were impaired. This is indicative of the indispensable role played by the Wave2/Abi1 complex in MK maturation and lamellipodia formation [46, 49].

It is known that the genetic mutations in some mouse models fully recapitulate the phenotype of the corresponding human disease. This is not always the case, however. In fact, mutation or deletion of several genes (e.g., *NFE2*, *Wave2*, and *Spectrin*) known to be important for thrombopoiesis in mouse models, have not been identified in patients with thrombocytopenia [8, 46, 50]. Consequently, we are unable to validate the function of these genes in human cells. Fortunately, recent developments with human ESCs [51] and iPSCs [52–54] and with gene-targeting technology enable us to provide an alternative tool for the study of human hematopoietic ontology and lineage commitment. For example, we used gene manipulation with human iPSCs to characterize the role played by *c*-*MYC* in human megakaryopoiesis. It had already been reported that *c*-*Myc* plays an essential role in both embryonic and adult hematopoiesis, though its effects on megakaryopoiesis and thrombopoiesis in various mouse models remained unclear [55–58]. Two studies of the effects of inducible *c*-*Myc* overexpression on the control of MK-specific differentiation showed that *c*-*Myc* exerts a positive effect on the proliferation of MK progenitors [55, 56]. Moreover, *c*-*Myc* is reportedly essential for the TPO-c-mpl axis in megakaryopoiesis [57]. On the other hand, more recent studies using *c*-*Myc*-deficient mice showed that the absence of the gene actually leads to a cell-autonomous increase in the number of low-ploidy MKs and platelets [58]. The reasons for this apparent discrepancy are still not completely clear. However, by comparing the efficiency of MK and platelet generation from several iPS clones established using retroviral vectors harboring either four (*OCT3/4*, *SOX2*, *KLF4* and *c*-*MYC*) or three (without *c*-*MYC*) reprogramming factors, we demonstrated that although *c*-*MYC* is beneficial for MK proliferation, its sustained expression inhibits platelet release. This idea was confirmed by additional experiments showing that overexpression of *c*-*MYC*, but none of the other reprogramming factors (*OCT3/4*, *SOX2*, *KLF4*), recapitulated the time course of the enhanced megakaryopoiesis. In addition, flow cytometry revealed that most of the cells in specimens overexpressing *c*-*MYC* were CD41a+GPIbα+, but cells in the other specimens were not, though only mononuclear and lower-ploidy cells were present. The numbers of platelets per MK, one indicator of MK maturation, were quite low in specimens overexpressing *c*-*MYC*, and the released platelet-like particles did not express GPIbα, suggesting that MKs from specimens overexpressing *c*-*MYC* were immature. Interestingly, the phenotype of platelets generated from MKs overexpressing *c*-*MYC* was similar to that in *Gata1*−/− mouse models, which showed low expression of GPIbα, abnormal ultrastructure, and defective integrin αIIbβ3 activation in response to thrombin or ADP [59].

**Fig. 2:**
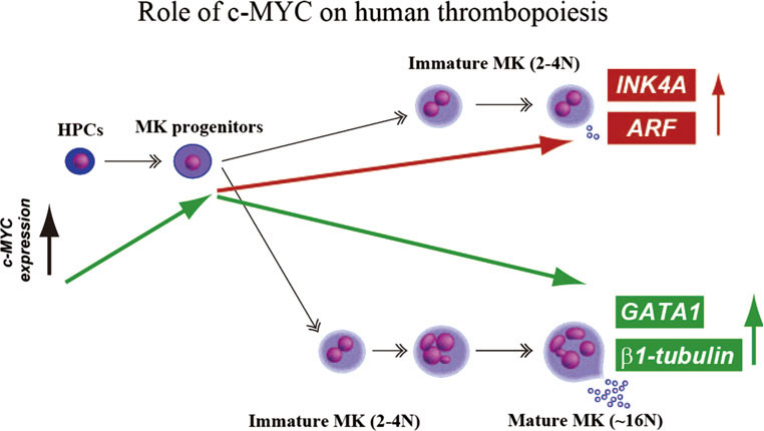
Proposed model of thrombopoiesis by *c*-*MYC*. We observed at least two distinct patterns of megakaryopoiesis in association with* c-MYC* kinetics. One is that excessive and sustained expression of *c*-*MYC* in MKs induces *INK4A* and *ARF*, leading to senescence and apoptosis without maturation of MKs. Another is that a decline in the *c*-*MYC* expression after a transient increase is a hallmark for MKs that production of functional GPIbα+ platelets is apparently detectable

The results summarized above led to us to conclude that excessive and sustained expression of *c*-*MYC* in human ESCs may promote lineage commitment into megakaryopoiesis without maturation. Moreover, overexpression of *c*-*MYC* in hematopoietic cells to levels that are inhibitory for platelet generation activates expression of the senescence/apoptosis-inducing *p14 (ARF)* and *p16 (INK4A)* genes, leading to senescence and apoptosis without maturation (Fig. 2). Thus, there appears to be a narrow window of *c*-*MYC* expression for efficient platelet development.

To further confirm whether an increase and subsequent decline in *c*-*MYC* is critical for megakaryopoiesis leading to an efficient platelet yield, we prepared a Sendai viral vector (SeV) harboring the four reprogramming genes; this enabled RNA viral transduction during the generation of human iPSCs without integration of DNA into the chromosome [60]. Thereafter, a doxycycline (DOX)-inducible *c*-*MYC* overexpression system in a lentiviral vector was applied to the SeV-based human iPSCs. This approach enabled us to confirm that transient up-regulation of *c*-*MYC* expression at the level of the MK progenitors and its subsequent decline increased the total numbers of mature MKs, proplatelets and GPIbα+ platelets [13].

### Studying thrombopoiesis using patient-specific iPSCs

**Fig. 3:**
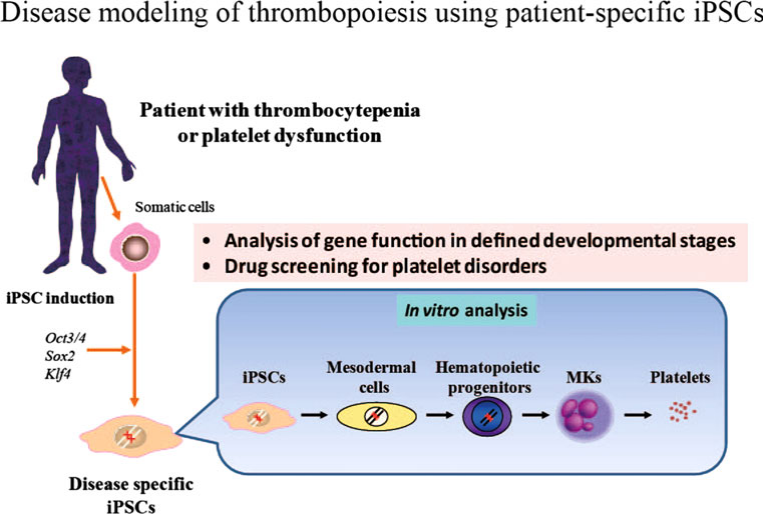
Disease modeling of thrombopoiesis using patient-specific iPSCs. iPS cell technology enables us to model human disease in vitro. Disease-specific iPSCs can be established from somatic cells in an individual patient by introduction of the defined reprogramming factors. Combing with in vitro differentiation system, genetic and epigenetic studies would reveal the pathogenesis of impaired megakaryocyte development in defined developmental steps. Novel drug screening for platelet disorders will also be applicable by using this system

Our culture system enables us to investigate in detail the developmental stages of MKs and platelets derived from human iPSCs. Moreover, MKs derived from disease-specific human iPSCs represent a powerful tool for investigating the unresolved aspects of the mechanisms underlying thrombocytopenia and for screening novel therapeutic agents for patients suffering from reduced platelet production and/or their impaired function (Fig. 3). This is illustrated in the following example. Congenital amegakaryocytic thrombocytopenia (CAMT) is an autosomal recessive disorder caused by the loss of function of *c*-*MPL*, the gene encoding the TPO receptor. It presents at birth with severe thrombocytopenia and evolves into fatal bone marrow failure in the early years of life [61]. A *c*-*Mpl*−/− mouse model failed to fully recapitulate the disease phenotype; *c*-*Mpl*−/− mice showed sustained thrombocytopenia and reduced numbers of marrow cells throughout their lives, but they survived to old age without developing bone marrow failure. To clarify the differential phenotype caused by the loss of *MPL* between humans and mice, we established iPSCs derived from skin fibroblasts collected from a CAMT patient treated with curative bone marrow transplantation. We then used our in vitro culture system to obtain hematopoietic progenitors to evaluate the function of *MPL* during the early and late phases of human hematopoiesis [62]. This patient’s iPSCs provided valuable insight into the mechanism of CAMT development.

### Application of human PSC-derived platelets for future transfusion medicine

#### Proof of concept: the stable supply of HLA-identical platelet concentrates

It is well known that repeated transfusion induces production of antibodies against allogenic HLA on the transfused platelets [63], which renders the patient unresponsive to platelet transfusion therapy. To avoid such immune reactions following multiple allogeneic platelet transfusions, it is desirable to use HLA-matched or autologous platelets. In this context, we propose that the use of HLA-matched human iPSCs may be an effective strategy for generating functional platelets for patients requiring repeated transfusion. We have shown that iPSC-derived platelets are functional in vivo and could represent a novel solution for the shortage of donated platelets [13]. Now we attempt to modify the culture system to provide huge amount of platelets for clinical application.

#### Preservation of platelet function in vitro using metalloproteinase inhibitor

The functional platelet paradigm in hemostasis and thrombosis is the initiation of platelet adhesion to the extracellular matrix [64]. Key events in that process are the interaction between GPIbα and vWF present in the extracellular matrix [64], as well as the simultaneous interaction between surface-bound collagen and the platelet receptors GPVI and integrin α2β1 [65]. The net result is activation of integrin αIIbβ3, which turns into a competent fibrinogen receptor and leads to the formation of platelet aggregates [64]. Notably, aged platelets show a tendency to shed GPV and the extracellular domain of GPIbα, which contains the binding sites for vWF and thrombin [66, 67]. This phenomenon makes it difficult to preserve platelet concentrates for more than 7 days.

The shedding process likely involves the action of ADAM 17 and leads to a reduction in platelet function [42, 68]. We observed that, in culture, mouse ESC-derived platelets shed the extracellular domains of GPIbα and GPV, as well as GPVI, possibly through the action of ADAM10, which reduces αIIbβ3 activation and actin polymerization and impairs thrombus formation. However, administration of the metalloproteinase inhibitor GM6001 during differentiation increased the expression of GPIbα and GPVI and improved both thrombogenesis in vitro and post-transfusion recovery in vivo [25]. Administration of GM6001 also inhibited shedding of the extracellular domain of GPIbα from human iPSC-derived platelets [13]. These data suggest that regulation of metalloproteinases in culture could be useful for obtaining high-quality and efficacious human iPSC-derived platelets for transfusion.

## Conclusions and future directions

Several gene-targeted mouse models have provided important information about thrombopoiesis. While the genetic interventions, i.e., mutations by mouse models recapitulate the phenotype of the corresponding human disease, some of the models fail to show the mirror of human diseases. Of note from recent important work is that there is the difference of ortholog-matched mRNA expression between mouse and human platelets by using RNA sequence technology. This study showed that 4,990 mRNAs were commonly expressed both in human and mouse platelets, but 3,592 mRNAs were specific for human platelets and 1,022 mRNAs were specific for mouse platelets. They are associated with previously described functional disparities between mouse and human platelets at the transcript level, including protease activated receptor-1 (PAR1) [36, 69], protease activated receptor-3 (PAR3) [70], and platelet activating factor receptor (PAF-R) [71]. These strongly indicate the value of the study regarding human thrombopoiesis by human hematopoietic cells. In general, human primary hematopoietic stem/progenitor cells derived from cord blood, bone marrow, or peripheral blood are a main resource. We herein propose human PSCs as an alternative source.

MKs generated from human PSCs in vitro have the potential to form proplatelets that function like those derived from human HSCs, and the platelets released are functionally comparable to human peripheral blood-derived platelets. The in vitro generation of MKs and platelets from human PSCs has four advantages over primary HSCs. First, human PSCs are pluripotent cells that can proliferate almost indefinitely in vitro, thereby stably providing experimental samples that yield reproducible results. By contrast, inescapable variation in genetic background for each experiment makes it difficult to obtain consistent results with primary HSCs derived from cord blood or bone marrow. Second, human PSCs are a promising cell source for studying the ontogeny of blood cells in defined steps. It has been well demonstrated in mouse models that HSCs can provide all types of blood cells throughout life. Blood cells arise at the yolk-sac and transit to the AGM, fetal liver, and bone marrow during ontogeny [72–74]. In humans, however, the development of hematopoietic cells has not been characterized because of the difficulty in obtaining samples from human embryos. Human PSCs are an effective tool that enables one to recapitulate human ontology in vitro by appropriately defining the culture conditions, as we have shown. Third, human PSCs are easily amenable to gene manipulation.

Megakaryopoiesis is a complex process governed by the orchestrated activities of numerous molecular mediators, especially transcriptional factors, signaling molecules, microRNAs, and the products of their various target genes. Many transcriptional factors including Ets1, Runx1, Scl/tal1, Gata1, NFE2, Mef2C, MafG, and MafK are involved in megakaryopoiesis [75–77].

Megakaryopoiesis is also controlled by a lot of microRNAs by regulating the expression of these target genes [71]. To study the unknown role of transcriptional factors or microRNAs on human thrombopoiesis, human PSCs should be a useful tool. For example, we were able to demonstrate the novel function of *c*-*MYC* on human thrombopoiesis by using in vitro differentiation of human PSCs with gene manipulation.

Fourth, patient-specific iPSCs facilitate the study of disease mechanisms and drug screening [78]. Although gene-targeted mouse models have provided important information on the pathogenesis of human disease, some human diseases are not completely recapitulated in these models. For example, to clarify the function of *c*-*MPL*, the TPO receptor, we established iPSCs from skin fibroblasts collected from a patient with CAMT. In vitro differentiation of CAMT-iPSCs revealed the critical role played by *c*-*MPL* during early human thrombopoiesis. We anticipate that future technical advances in genome sequencing and/or epigenome analysis will enable identification of numerous gene mutations associated with congenital thrombocytopenia or megakaryocytic leukemia. When that happens, disease-specific iPSCs would be a powerful tool for distinguishing pathogenic gene mutations from the many neutral mutations. Finally, establishment of an in vitro system for platelet generation from human iPSCs could usher in a new era of platelet transfusion therapy. Platelets generated from human iPSCs in vitro show normal function during thrombus formation in living mice [13], but a more efficient system for platelet generation will be required for future clinical applications.
